# Antibiotic Susceptibility, Clonality, and Molecular Characterization of Carbapenem-Resistant Clinical Isolates of *Acinetobacter baumannii* from Washington DC

**DOI:** 10.1155/2020/2120159

**Published:** 2020-07-09

**Authors:** Garima Bansal, Rachelle Allen-McFarlane, Broderick Eribo

**Affiliations:** Howard University, Department of Biology, 415 College St. NW., Washington, DC 20059, USA

## Abstract

The occurrence of carbapenem-resistant (CR) strains of *Acinetobacter baumannii* is reported to contribute to the severity of several nosocomial infections, especially in critically ill patients in intensive care units. The present study aims to determine the antibiotic susceptibility, clonality, and genetic mechanism of carbapenem resistance in twenty-eight *Acinetobacter baumannii* isolates from four hospitals in Washington DC. The antibiotic susceptibility of the isolates was determined by VITEK 2 analyses, while PCR was used to examine the presence of antibiotic-resistant genes and mobile genetic elements. Trilocus multiplex-PCR was used along with pulsed-field gel electrophoresis (PFGE) for strain typing and for accessing clonal relationships among the isolates. Antimicrobial susceptibility testing indicated that 46% of the isolates were carbapenem-resistant and possessed MDR and XDR phenotypes. PFGE clustered the 28 isolates into seven clonal (C1–C7) complexes based on >75% similarity cut-off. Thirty-six percent of the isolates belonged to international clone II, while 29% were assigned to Group 4 by trilocus multiplex-PCR. Although the *bla*_*OXA-51-like*_ gene was found in all the isolates, only 36% were positive for the *bla*_*OXA-23-like*_ gene. PCR analysis also found a metallo-*β*-lactamase (MBL) gene (*bla*_*VIM*_) in 71% of the isolates. Of the 13 CR isolates, 8 were PCR positive for both *bla*_*VIM*_ and *bla*_*OXA-23-like*_ genes, while 5 harbored only *bla*_*VIM*_ gene. This study revealed the emergence of VIM carbapenemase-producing *A*. *baumannii* isolates, which has not been previously reported in the United States.

## 1. Introduction

*Acinetobacter baumannii* has emerged as an important nosocomial pathogen associated with hospital-acquired infections worldwide [1–3]. Multidrug-resistant (MDR) *A*. *baumannii* strains are associated with infections such as ventilator-associated pneumonia, septicemia, urinary tract, soft skin, wound, and meningitis, especially in immunocompromised patients in ICU settings [4–7]. The success of *A*. *baumannii* as an emerging nosocomial pathogen is mostly due to its efficiency in acquiring new antibiotic-resistant determinants [8, 9] which may have contributed to the high antibiotic index as classified by the World Health Organization (WHO). The carbapenem-resistant *A*. *baumannii* is now grouped among the leading causes of bacterial nosocomial infections throughout the world. This special category of pathogens termed “ESKAPE” consists of *Enterococcus faecium*, *Staphylococcus aureus*, *Klebsiella pneumoniae*, *Acinetobacter baumannii*, *Pseudomonas aeruginosa*, and *Enterobacter spp*. The acronym ESKAPE was developed by culling the first letter of each of the genus names in the group [10].

Antibiotic resistance in *A*. *baumannii* is mediated by enzymatic degradation of antibiotics, mutations/modification of target sites, reduced expression of porins, and overexpression of multidrug efflux pumps [3, 11, 12]. However, resistance to carbapenem is often mediated by *β*-lactamases including carbapenem-hydrolyzing class D *β*-lactamases (CHDLs) and metallo-*β*-lactamases (MBLs). Resistance by class D *β*-lactamases (CHDLs), also known as oxacillinases, is mainly mediated by the production of carbapenemase enzymes encoded by genes of the *bla_OXA-23_*, *bla_OXA-40_*, and *bla_OXA-58_*-like lineage; however, *bla_OXA-23_* is reported to be the most prevalent worldwide [13, 14]. Transposable elements such as insertion sequences (*ISAba1*) have an important role in carbapenem resistance in *A*. *baumannii* and are present upstream at promoter regions of the *bla_OXA-23_*, *bla_OXA-40_*, *bla_OXA-58_*, and *bla_OXA-51_* genes causing overexpression of these resistant genes [15].

According to the Centers for Disease Control and Prevention (CDC) in 2013, 7,300 (63%) out of 12,000 annual infections in the USA were due to MDR carbapenem-resistant *A*. *baumannii*, leading to about 500 deaths annually [16]. By the late 1990s, carbapenems were the most important antimicrobial drugs of choice by clinicians as they represent the “last-line” drugs for the treatment of infections caused by MDR *A*. *baumannii* due to their high efficiency and low toxicity [17]. However, several reports have shown that carbapenem-resistant *Acinetobacter baumannii* (CRAB) strains are on the rise in infections among hospitalized patients in USA. According to a recent survey conducted in USA in 2016, resistance to carbapenems and colistin increased from 21.0% in 2003–2005 to 47.9% in 2009–2012, and from 2.8% in 2006–2008 to 6.9% in 2009–2012, respectively [18]. In another study by Cai et al., data collected from 206 acute care hospitals in USA in 2009–2013 revealed that *A*. *baumannii* had the highest carbapenem-resistant rate (blood 40.1%, respiratory tract 50.4%, urine 42.0%, and others 42%) compared to other three pathogens, namely, *Pseudomonas aeruginosa*, *Klebsiella pneumoniae*, and *Escherichia coli* [19].

Despite the worldwide reports on the clinical significance of *A*. *baumannii* infections and the associated increased morbidity and mortality, there are very few published reports on carbapenem-resistant *A*. *baumannii* in Washington DC. Therefore, the present study aims to determine the clonality, antibiotic susceptibility profiles, and resistance patterns of *A*. *baumannii* isolates from four hospitals within Washington DC.

## 2. Materials and Methods

### 2.1. Bacterial Isolates and Identification

A total of 28 clinical isolates of *Acinetobacter spp*. were randomly chosen from Washington DC public health laboratory for this study. These isolates were collected from four different hospitals in Washington DC during 2011–2014 time period. The isolates were obtained from different clinical sources as follows: sputum (3%), peritoneum (3%), urine (3%), respiratory tract (7%), trachea (7%), catheter (7%), blood (17%), and wounds (53%). The different age groups of the patients were as follows: 0–25 years (*n* = 3), 25–50 years (*n* = 6), 50–75 years (*n* = 15), and 75–100 years (*n* = 4), while the gender distribution of the patients was as follows: 67.5% men and 32.5% women.

All isolates were identified as *Acinetobacter baumannii* (ACB) complex using a VITEK^®^ 2 automated instrument ID system (Biomerieux, Inc., Hazelwood, MO). Isolates positive for *bla_OXA-51_* gene were identified as *A*. *baumannii* by PCR method. Isolates were stored at −20°C in trypticase soy broth (TSB) supplemented with 20% glycerol until further analysis could be performed.

### 2.2. Antimicrobial Susceptibility Testing

Antibiotic susceptibility testing was determined using the VITEK^®^ 2, (version 06.01) (Biomerieux, Inc., Hazelwood, MO) with cards AST-XN04 and AST-GN46 for the following eight antimicrobial categories: aminoglycosides (gentamicin, tobramycin), carbapenems (imipenem, doripenem), fluoroquinolones (ciprofloxacin, levofloxacin), extended-spectrum cephalosporins (ceftazidime, cefotaxime, cefepime, ceftriaxone), folate pathway inhibitor (trimethoprim/sulfamethoxazole), penicillin (piperacillin, ticarcillin), *β*-lactam/*β*-lactamase inhibitors combinations (ampicillin-sulbactam), and tetracyclines (tetracycline, tigecycline). Minimum inhibitory concentrations (MICs) were interpreted according to the Clinical and Laboratory Standards Institute guidelines [20]. The reference strains *Escherichia coli* ATCC 25922 and *Pseudomonas aeruginosa* ATCC 27853 were used as quality controls. Multidrug-resistance (MDR) was defined as isolates resistant to at least three categories of antimicrobial drug classes, whereas extensively drug-resistant (XDR) strains were resistant to all antibiotics categories except polymyxin (colistin and polymyxin B) and tigecycline [21].

### 2.3. Pulsed-Field Gel Electrophoresis (PFGE)

PFGE was performed according to the standard operating procedure for PulseNet PFGE of *Escherichia coli* O157:H7, *Escherichia coli* non-O157 (STEC), *Salmonella serotypes*, *Shigella sonnei*, and *Shigella flexneri* (http://www.cdc.gov/pulsenet/protocols.htm) with the following modifications: the genomic DNA in the agarose plugs was digested with *ApaI* (50 U/*μ*l; New England BioLabs, Ipswich, MA) for 3 hours at 37°C. The restricted genomic DNA fragments were separated in 1% SeaKem Gold Agarose gels in 0.5 × TBE buffer using CHEF mapper system (Bio-Rad Laboratories, Hercules, CA) at 14°C, 200 V (6V/cm), with initial pulse time of 2.2 s and final pulse time at 35 s. *Salmonella* strain 201 was digested with restriction enzyme *Xba I* (5 U/*μ*l) and used as a DNA standard. PFGE band patterns were analyzed by the BioNumerics software, version 6.6.4.0 (Applied Maths, St-Martens-Latem, Belgium), using Unweighted Pair Group Method and Arithmetic Mean (UPGMA) with 1.5% tolerance and 1.5% optimization. All isolates with similarity index of >75% were grouped together in the same cluster and showed similar PFGE banding pattern [22].

### 2.4. Trilocus Sequence Typing (3LST)

Trilocus sequence typing method was used to assign international/global clonal groups to the isolates as described by Turton et al. [23]. Briefly, multiplex-PCR was performed using primers targeting alleles of *ompA*, *csuE*, and *bla_OXA-51_*-like genes. Isolates were assigned to respective groups based on the different combinations of gene amplifications as described in [23, 24].

### 2.5. Phenotypic Metallo-*β*-Lactamase (MBL) Detection

Thirteen imipenem resistant isolates were screened for the presence of metallo-*β*-lactamases (MBLs). Briefly, Mueller-Hinton agar plates (BD, DIFCO™) were inoculated with test strains adjusted to McFarland 0.5 standard, and after drying the following tests were performed [25, 26].

#### 2.5.1. EDTA-Disk Synergy Test

Imipenem disk (BD BBL™, 10 *μ*g) and filter paper disk with 10 *µ*l of 0.5 M EDTA solution were placed 10 mm apart. The presence of enlarged/synergistic zone of enhancement was interpreted as metallo-*β*-lactamase (IMP or VIM type) positive strain.

#### 2.5.2. Combined Disk Diffusion Test

Three Imipenem (BD BBL™, 10 *μ*g) disks, one serving as control and the other two with 0.1 M EDTA (10 *μ*l) and 0.5 M EDTA (10 *μ*l), were placed 10 mm apart from each other on the Mueller-Hinton agar plates. After overnight incubation, increase of ≥4 mm in the zone diameters of imipenem disk with 0.1 M EDTA and increase of ≥7 mm in the zone diameters of imipenem disks with 0.5 EDTA were interpreted as a positive test for MBL producing strain.

### 2.6. Colony PCR for Detection of Carbapenemases Genes and Mobile Genetic Elements

Colony PCRs were performed to detect the presence of four main class D OXA *β*-lactamase genes (*bla_OXA–23-like_*, *bla_OXA–24-like_*, *bla_OXA–51-like_*, and *bla_OXA–58-like_*) and three class B, also known as metallo-*β*-lactamases, genes (*bla_IMP_*, *bla_VIM_*, and *bla_SIM_*). Isolates confirmed positive for oxacillinases genes were further screened for the presence of *ISAba1* element upstream of these genes [27]. Briefly, colony PCRs were performed in a 50 *μ*l reaction mixture with 25 *μ*l of EconoTaq PLUS GREEN 2X Master Mix (Lucigen, Middleton, WI), 0.5 *μ*l each of forward primer (100 *μ*M) and reverse primer (100 *μ*M), and 25 *μ*l of nuclease-free water. Small amount of bacterial colony was removed using the sterile toothpick and added to the master mix. Amplification was carried out under the following conditions: denaturation at 94°C (3 min), 35 cycles of denaturation at 94°C (45 s), annealing for 45 s as described previously [28], and extension at 72°C for 1 min, followed by a final extension of 72°C for 1 min. The Qiagen purification kit (Qiagen, USA) was used to purify amplified PCR products, and both strands were sequenced by automated AB13100 DNA sequencer (Applied Biosystems) system. The nucleotide sequences were analyzed using the basic local alignment search tool (BLAST).

## 3. Results

### 3.1. Antibiotic Susceptibility Profiles

In order to gain insight into the antibiotic susceptibility profile of the *A*. *baumannii* strains used in this study, sixteen antibiotics representing eight antimicrobial classes were used. The antimicrobial agents included tobramycin (18%), ampicillin/sulbactam (32%), gentamicin (32%), cefotaxime (36%), ceftriaxone (36%), cefepime (36%), ceftazidime (43%), tetracycline (43%), trimethoprim/sulfamethoxazole (50%), levofloxacin (54%), imipenem (46%), doripenem (46%), ciprofloxacin (57%), meropenem (57%), and amikacin (61%), respectively. All isolates were sensitive to tigecycline except isolates C4 and C24. Of the 28 isolates, 18% (*n* = 5) were multidrug-resistant (MDR) and 29% (*n* = 8) were extreme drug-resistant (XDR) based on criteria provided by Magiorakos and colleagues [21]. The antibiotic susceptibility results are summarized in Table 1.

### 3.2. Pulsed-Field Gel Electrophoresis (PFGE)

**Figure 1:**
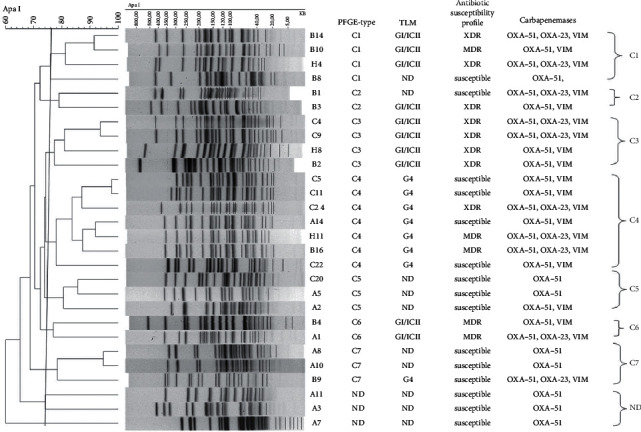
Dendrogram representing the PFGE pattern of 28 clinical *A*. *baumannii* isolates in comparison with trilocus sequence-based multiplex-PCR (TLM), antibiotic susceptibility, and carbapenemases. BioNumerics V6.01 platform used for analyzing the *ApaI*-digested PFGE fingerprints using Unweighted Pair Group Method and Arithmetic Mean (UPGMA) with 1.5% tolerance and 1.5% optimization. Seven clusters (C1–C7) were detected using cut-off value at >75% for the similarity coefficient (vertical line).

Pulsed-field gel electrophoresis (PFGE) was used to determine the genetic and clonal relationships among these clinical isolates using *ApaI*-digested DNA. PFGE analysis clustered the isolates into seven clonal complexes, C1–C7, using a cut-off value of >75% similarity (Figure 1). All isolates grouped in cluster C3 were carbapenem-resistant and possessed the XDR phenotype. Isolates in clusters C1, C2, C4, and C6 were XDR and/or MDR, except isolates B8, B1, C5, C1, A14, and C22. However, the isolates in clusters C5 and C7 were susceptible to all the antibiotics tested. The largest cluster identified as C4 included seven isolates, out of which two (B16 and H11) were MDR and one (C24) possessed an XDR phenotype. Isolates A11, A3, and A7 could not be assigned to any clonal group by this method.

### 3.3. Trilocus Sequence-Based Typing (3LST)

**Figure 2:**
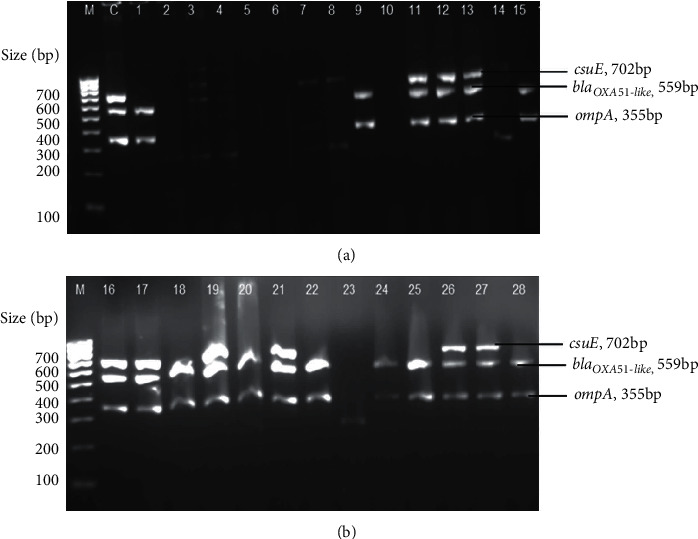
Multiplex-PCR amplification for *ompA*, *csuE*, and *bla_OXA-51-like_* alleles for Group 1 primers. Lane C is a control *A*. *baumannii* ATCC 19606 strain. Lanes 1-28 represent isolates A1, A2, A3, A5, A7, A8, A10, A11, A14, B1, B2, B3, B4, B8, B9, B10, B14, B16, C4, C5, C9, C11, C20, C22, C24, H4, H8, and H11, respectively. M is a 100 bp size marker ladder (HyperLadder™, Bioline).

A 3LST-based PCR typing method, targeting three genes (*ompA*, *csuE*, and *bla_OXA-51-like_*), was employed to assign the global clonal lineage of the isolates. The gel image of PCR amplification for *ompA*, *csuE*, and *bla_OXA-51-like_* alleles for all the isolates tested for Group 1 primers is shown in Figure 2; however, there was no amplification observed for Group 2 primers (data not shown). Of the 18 isolates typed by this method, 10 (36%) were assigned to Group 1 or international clone II lineage (G1/ICII), while the remaining eight were assigned to Group 4, based on the proposed schemes [23, 24]. Ten isolates could not be assigned to any international clonal group due to lack of amplification in the PCR reaction. However, all the isolates (*n* = 10) belonging to the G1/ICII group were carbapenem-resistant and exhibited either a MDR and/or XDR phenotype, whereas most of the isolates (*n* = 5) in Group 4 were susceptible to the tested antibiotics, and only three were carbapenem-resistant (Table 1 and Figure 1).

### 3.4. Identification of Class B and D *β*-Lactamases Genes

**Figure 3:**
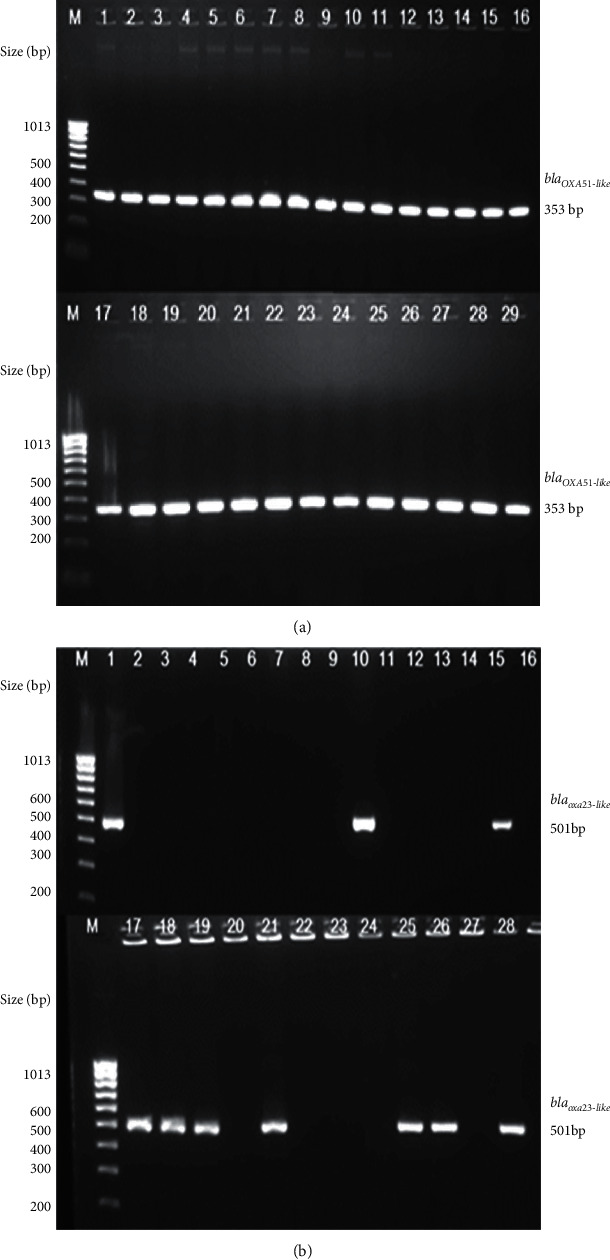
(a) Colony PCR amplification for *bla_OXA-51-like_* carbapenemase gene. Upper and lower panel: Lane 1 is a positive control *A*. *baumannii* ATCC 19606 strain. Lanes 2-29 represent isolates A1, A2, A3, A5, A7, A8, A10, A11, A14, B1, B2, B3, B4, B8, B9, B10, B14, B16, C4, C5, C9, C11, C20, C22, C24, H4, H8, and H11, respectively. M is a 100 bp size marker ladder (HyperLadder™, Bioline). (b) Colony PCR amplification for *bla_OXA-23-like_* carbapenemase gene. Upper and lower panel: Lanes 1–28 represent isolates A1, A2, A3, A5, A7, A8, A10, A11, A14, B1, B2, B3, B4, B8, B9, B10, B14, B16, C4, C5, C9, C11, C20, C22, C24, H4, H8, and H11, respectively. M is a 100 bp size marker ladder (HyperLadder™, Bioline).

**Figure 4:**
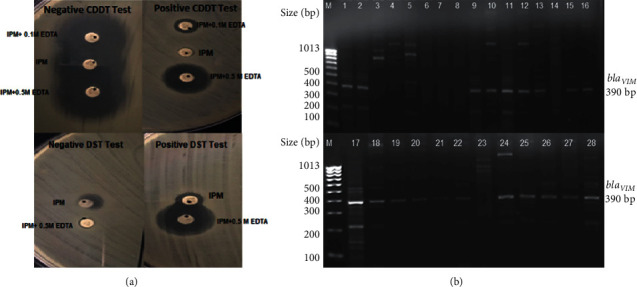
(a) Phenotypic tests to detect MBL production. The upper left panel shows the negative combined disk diffusion test (CDDT) and the upper right panel is a positive CDDT with increase in zone diameters of imipenem disks (≥4 with imipenem/0.1 M EDTA and ≥7 with imipenem/0.5 M EDTA). The lower left panel shows a negative EDTA-disk synergy test (DST) while the lower right panel shows a positive DST with a synergistic inhibition zone between imipenem disk and imipenem with 0.5 M EDTA. (b). Colony PCR amplification for blaVIM gene. Lanes 1-28 represent isolates A1, A2, A3, A5, A7, A8, A10, A11, A14, B1, B2, B3, B4, B8, B9, B10, B14, B16, C4, C5, C9, C11, C20, C22, C24, H4, H8, and H11, respectively. M is a 100 bp size marker ladder (HyperLadder™, Bioline).

All the clinical isolates (*n* = 28) were PCR positive for the *β*-lactamases gene *bla_OXA-51-like_* (Figure 3(a)). Additionally, 10 out of 28 isolates (36%) also harbored the class *D OXA β-lactamase* gene *bla_OXA–23-like_* (Figure 3(b)). None of the isolates harbored other CHDLs, such as *bla_OXA–24-like_* and *bla_OXA–58-like_* genes. Class B metallo-*β*-lactamases gene *bla_VIM_* was detected in 71% (*n* = 20) of the isolates (Figure 4(b)); however, other metallo-*β*-lactamase genes, such as *bla_IMP_* and *bla_SIM_*, were not detected in any of the isolates.

### 3.5. Phenotypic Metallo-*β*-Lactamase (MBL) Detection

All 13 imipenem resistant isolates were found to be positive for EDTA-disk synergy and combined disk diffusion tests (Figure 4(a)).

### 3.6. Mobile Genetic Elements

**Figure 5:**
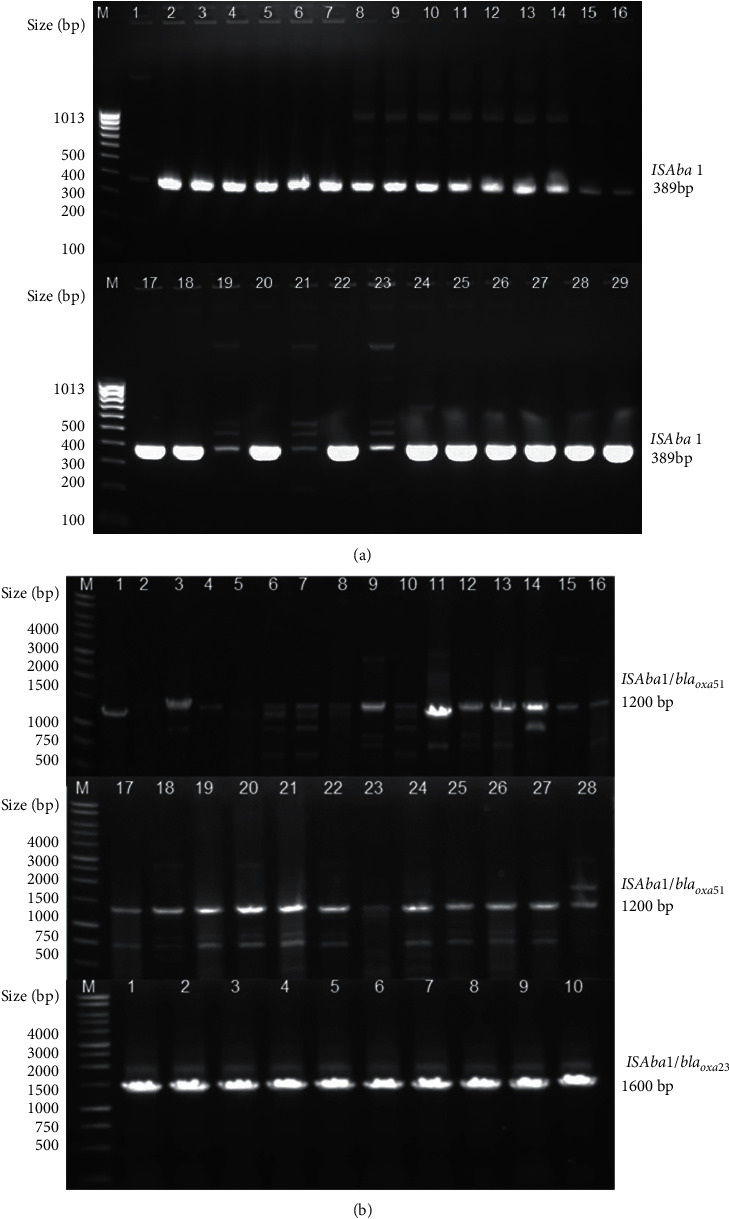
(a) Colony PCR amplification for insertion sequence (*ISAba1*) gene. Upper and lower panel: Lane 1 is a control *A*. *baumannii* ATCC 19606 strain. Lanes 2-29 represent isolates A1, A2, A3, A5, A7, A8, A10, A11, A14, B1, B2, B3, B4, B8, B9, B10, B14, B16, C4, C5, C9, C11, C20, C22, C24, H4, H8, and H11, respectively. (b) PCR amplification for *ISAba1* forward primer and *bla_OXA-51-like_* gene reverse primers *ISAba1*F/OXA-51R. Lanes 1-28 (except Lane 5) represent isolates A1, A2, A3, A5, A8, A10, A11, A14, B1, B2, B3, B4, B8, B9, B10, B14, B16, C4, C5, C9, C11, C20, C22, C24, H4, H8, and H11, respectively. The lower panel represents amplification for *ISAba1* forward primer and *bla_OXA-23-like_* gene reverse primers *ISAba1*F/OXA-23R. Lanes 1-10 represent isolates A1, B1, B9, B14, B16, C4, C9, C24, H4, and H11, respectively.

All isolates were positive for the presence of insertion sequence *ISAba1* (Figure 5(a)). The insertion sequence *ISAba1* was located upstream in all *bla_OXA–23-like_* gene positive isolates, whereas it was present in 96% of *bla_OXA-51-like_* gene carrying isolates (Figure 5(b)).

## 4. Discussion

Based on the recent criteria by World Health Organization (WHO), carbapenem-resistant *A*. *baumannii* (CRAB) is among the top priority pathogens [29]. There are several reports of the emergence and rise of carbapenem-resistant *A*. *baumannii* strains in hospitals throughout United States [22, 28, 30, 31], but limited information is available regarding the genetic basis of hospital-acquired carbapenem resistance in Washington DC region. To address this research gap, twenty-eight nosocomial strains of *A*. *baumannii* were included in this study.

With regard to antibiotic susceptibility, the data revealed that 13 (46.4%) of the 28 isolates were carbapenem-resistant. Out of 13 carbapenem-resistant isolates, 5 (38%) were MDR, while 8 (61%) were of XDR phenotype (Table 1). The presence of XDR isolates is of great concern, since infections caused by XDR isolates are difficult to treat because they are not susceptible to the known commercially available drugs and, thus, pose considerable infection control issues [32]. In a recent study from the Harbor-UCLA Medical Center (HUMC), California, USA, 57% of 21 isolates of *A*. *baumannii* tested for antibiotic susceptibility were found to be carbapenem-resistant; and all isolates were sensitive to colistin and tigecycline; however, none of the isolates possessed an XDR phenotype. Similarly, there has been an increase in reports of carbapenem-resistant *A*. *baumannii* from hospitals in other regions of the United States [22, 28, 30, 31].

Several genotypic methods are available for surveillance, strain typing, and epidemiological studies of *A*. *baumannii* outbreaks [33]; however, there is not a single ideal method. In this study, we used pulsed-field gel electrophoresis (PFGE) and trilocus sequence-based typing (3LST) methods for finding the genetic relatedness among the isolates. PFGE is a widely used method and is still considered as the “gold standard” for genotyping irrespective of the limitation of the interlaboratory reproducibility; on the other hand, 3LST method has an added advantage of providing information pertaining to pathogenicity [23, 34]. The genomic profile of isolates determined by PFGE appears to be in correlation with the antibiotic susceptibility pattern with few exceptions. Isolates with similar antibiotic susceptibility were clustered together in their respective clonal complexes, suggesting that they might be closely related and changes in the genome could be possibly linked to their phenotype. All isolates with similar antibiotic susceptibility profile were grouped into three clonal complexes, C1 (except isolates B10 and B8), C2 (except isolate B1), and C3, and were defined as XDR. Similarly, isolates in cluster C4 (except isolates C5, C11, A14, and C22) and C6 possessed MDR phenotypes. The remaining isolates grouped in clusters C5 and C7 were susceptible to all of the tested antibiotics (Figure 1 and Table 1).

Results from both PFGE and 3LST genotyping methods were in agreement (Figure 1). All isolates (*n* = 10) belonging to clusters C1(except isolate B8), C2 (except B1), C3, and C6 were assigned to G1/ICII category, while all isolates in cluster C4 belong to Group 4. Isolates in clusters 5 and 7 (except B9) could not be assigned to any global lineage. Similarly, isolates A11, A3, and A7 could not be genotyped by both methods. The analysis revealed that the largest clonal complex C4 (*n* = 7) included isolates from all the four hospitals irrespective of the origin. The results from the strain typing indicated that the isolates (*n* = 10) assigned to G1/ICII lineage were carbapenem-resistant and possessed MDR and/or XDR phenotypes. Recently, Warner and coworkers examined 38 clinical isolates of *A*. *baumannii* from two hospital outbreaks in Los Angeles County. In their report, nine isolates were identified as belonging to G1/ICII lineage and were typed as sequence type 2 (ST2) isolates using the Pasteur MLST scheme [30].

Regarding the mechanisms of resistance, there seems to be a general consensus that the enzymatic modification or degradation of *β*-lactams by different *β*-lactamases is a major mechanism for carbapenem resistance in *A*. *baumannii* isolates from different parts of the world [3, 14, 35–39]. The screening for OXA-type *β*-lactamases confirmed the presence of intrinsic chromosomally located OXA-51-like gene in all of the *A*. *baumannii* isolates in this study (Figure 3(a)). Additionally, OXA-23-like gene was detected in 36% (*n* = 10) of the isolates (Figure 3(b)). All OXA-23 positive strains were carbapenem-resistant and associated with insertion sequences *ISAba1* element present upstream in the promoter region of the gene, which may amplify the overexpression of the OXA-23 gene (Figure 5(b)).

The detection of VIM metallo-*β*-lactamases gene is noteworthy in this study. Thirteen out of twenty VIM-harboring isolates were carbapenem-resistant. The coexistence of OXA-23-like and VIM metallo-*β*-lactamases gene in eight isolates may have possibly contributed to carbapenem resistance in these isolates. Interestingly, OXA-23-like gene was not detected in the remaining five carbapenem-resistant isolates, B2, B3, B4, B10, and H8. Among these isolates, three (B2, B3, and H8) were resistant to agents in all eight antimicrobial categories and exhibited XDR phenotype, while isolates B4 and B10 were of MDR phenotype. This indicates that VIM metallo-*β*-lactamases might be the main contributor to carbapenem resistance in these five isolates. This is in contrast to the reports on *A*. *baumannii* outbreak investigation in the United States, where the expression of OXA-23-like gene was mainly attributed to carbapenem resistance [22, 28, 30, 31, 40–42].

This study presents the antibiotic susceptibility and molecular analysis of the carbapenem-resistant isolates of *A*. *baumannii* strains collected from four Washington DC hospitals. PFGE and TLST data suggest that the carbapenem-resistant strains mainly belonged to international clonal II lineage. To the best of our knowledge, this is the first report on the emergence of VIM metallo-*β*-lactamases gene in *A*. *baumannii* isolates in the USA. The data revealed prevalence of mobile genetic elements like insertion sequence (*ISAba1*) in all of the isolates. The results of this study suggest the need for continuous surveillance for carbapenem-resistant strains in Washington DC hospitals and the maintenance of proper infection control measures to prevent transmission of MDR and/or XDR strains.

## Figures and Tables

**Table 1:** Antibiotic susceptibility profiles of *Acinetobacter baumannii* isolates.

Isolate	Aminoglycosides		Carbapenems		Fluoroquinolones		Extended-spectrum cephalosporins				Folate pathway inhibitors	Penicillin		*β*-Lactam/*β*-lactamase inhibitors combinations	Tetracyclines	
	GM	TM	IPM	DOR	CIP	LEV	CTX	CRO	CAZ	FEP	SXT	PIP	TIC	SAM	TGC	TE
A1	S	S	**R**	**R**	**R**	**R**	**R**	**R**	**R**	**R**	**R**	**R**	**R**	S	_I_	_**R**_
A2	S	S	S	S	S	S	S	I	S	S	S	S	S	S	_S_	_S_
A3	S	S	S	S	S	S	I	I	I	S	S	S	I	S	_S_	_**R**_
A5	S	S	S	S	S	S	I	I	S	S	S	S	S	S	_S_	_S_
A7	S	S	S	S	S	S	S	S	S	S	S	S	S	S	_S_	_S_
A8	S	S	S	S	S	S	S	I	S	S	S	S	S	S	_S_	_S_
A10	S	S	S	S	S	S	S	I	S	S	S	S	S	S	_S_	_S_
A11	S	S	S	S	S	S	S	I	S	S	S	S	S	S	_S_	_S_
A14	I	S	S	S	**R**	**R**	I	I	S	I	**R**	S	S	S	_S_	_S_
B1	S	S	S	S	S	S	S	I	S	S	S	S	S	S	_S_	_S_
B2	**R**	**R**	**R**	**R**	**R**	**R**	**R**	**R**	**R**	**R**	**R**	**R**	**R**	**R**	_I_	_**R**_
B3	**R**	I	**R**	**R**	**R**	**R**	**R**	**R**	**R**	**R**	**R**	**R**	**R**	**R**	_I_	_**R**_
B4	I	**R**	**R**	**R**	**R**	I	**R**	**R**	**R**	I	S	**R**	**R**	S	_S_	_**R**_
B8	S	S	S	S	S	S	I	I	S	S	S	S	I	S	_S_	_S_
B9	S	S	S	S	S	S	S	I	S	S	S	S	S	S	_S_	_S_
B10	S	I	**R**	**R**	**R**	**R**	**R**	**R**	**R**	I	**R**	**R**	**R**	**R**	_S_	_**R**_
B14	**R**	I	**R**	**R**	**R**	**R**	**R**	**R**	**R**	**R**	**R**	**R**	**R**	**R**	_I_	_**R**_
B16	I	S	**R**	**R**	**R**	**R**	I	I	**R**	I	**R**	**R**	**R**	S	_I_	_I_
C4	**R**	**R**	**R**	**R**	**R**	**R**	**R**	**R**	**R**	**R**	**R**	**R**	**R**	**R**	_**R**_	_**R**_
C5	S	S	S	S	**R**	**R**	S	S	S	I	S	S	S	S	_S_	_S_
C9	**R**	I	**R**	**R**	**R**	**R**	**R**	**R**	**R**	**R**	**R**	**R**	**R**	**R**	_I_	_**R**_
C11	S	S	S	S	**R**	**R**	I	I	S	S	S	S	S	S	_S_	_S_
C20	S	S	S	S	S	S	S	I	S	S	S	S	S	S	_S_	_S_
C22	S	S	S	S	S	S	I	I	S	S	**R**	S	S	S	_S_	_S_
C24	**R**	S	**R**	**R**	**R**	**R**	I	I	I	**R**	**R**	**R**	**R**	**R**	_**R**_	_**R**_
H4	**R**	**R**	**R**	**R**	**R**	**R**	**R**	**R**	**R**	**R**	**R**	**R**	**R**	**R**	_I_	_**R**_
H8	**R**	**R**	**R**	**R**	**R**	**R**	**R**	**R**	**R**	**R**	**R**	**R**	**R**	**R**	_I_	_**R**_
H11	**R**	S	**R**	**R**	**R**	**R**	I	S	**R**	**R**	**R**	**R**	**R**	I	_I_	_I_

Isolates were designated as susceptible (S), intermediate (I), or resistant (R) according to CLSI guidelines. GM: gentamicin; TM: tobramycin; IPM: imipenem; DOR: doripenem; CIP: ciprofloxacin; LEV: levofloxacin; CTX: cefotaxime; CRO: ceftrioxone; CAZ: ceftazidime; FEP: cefepime; SXT: trimethoprim/sulfamethoxazole; PIPpiperacillin; TIC: ticarcillin; SAM: ampicillin/sulbactam; TGC: tigecycline; TE: tetracycline.

## Data Availability

The data used to support the findings of this study are included within the article, and any additional data are available from the corresponding author upon request.
